# Motor Capabilities and Body Composition in Health vs. Non-Health University Students: A Pilot Study

**DOI:** 10.3390/life15101504

**Published:** 2025-09-24

**Authors:** Ivana Živoder, Vesna Hodić, Nikolina Zaplatić Degač, Jasminka Potočnjak, Marija Arapović, Anica Kuzmić, Željko Jeleč, Goran Knežević, Mateja Znika, Tomislav Meštrović

**Affiliations:** 1 University Center Varaždin, Department of Physiotherapy, University North, 42000 Varazdin, Croatia; vesna.hodic@unin.hr (V.H.); nzaplatic@unin.hr (N.Z.D.); jpotocnjak@unin.hr (J.P.); marapovic@unin.hr (M.A.); akuzmic@unin.hr (A.K.); mznika@unin.hr (M.Z.); tmestrovic@unin.hr (T.M.); 2 Special Hospital St. Katarina, 10000 Zagreb, Croatia; zjelec@yahoo.com; 3 StatVall, 33000 Virovitica, Croatia; statistika.analiza@gmail.com

**Keywords:** students, motor ability, body analysis, body mass index, physical activity

## Abstract

Motor skills in students can be developed or improved through regular physical activity such as walking in nature, Nordic walking, hiking, cycling and swimming. This study aimed to examine the relationship between motor skills and various anthropometric and health-related factors, and to appraise any differences in motor performance and body mass index (BMI) on a sample of university students from Croatia. A total of 122 students (73 of them in health programs and 49 in non-health programs) aged 18 to 44 years participated in the study. Motor abilities were evaluated using standardized motor tests, while body composition was assessed via bioelectrical impedance analysis, which measured fat tissue, muscle and bone mass, metabolic age, degree of obesity, total body water, and BMI. While the groups were similar in terms of BMI and weight, students in non-health-related study programs had significantly higher values across a wide array of detailed body composition measures, particularly related to fat and muscle mass. Significant negative correlations were observed between body fat percentage and trunk lift performance (r = −0.55, *p* < 0.01), as well as between metabolic age and trunk lift performance (r = −0.44, *p* < 0.01) in health students. In non-health students, the strongest negative correlation was found between body fat percentage and flexibility (r = −0.47, *p* < 0.01). Higher muscle mass was a positive predictor of motor performance while higher fat mass and metabolic age were significant negative predictors. These findings underscore the impact of body composition on motor performance, particularly strength and flexibility, and highlight the need for targeted preventive strategies among university students. The study supports the implementation of early interventions promoting physical activity and healthy body composition to preserve motor abilities and long-term functional health in this critical age group—especially since lifestyle habits formed during university years tend to persist into adulthood.

## 1. Introduction

Motor skills, physical fitness and body composition in young adults [1] are key indicators of general health and well-being [2], and their dynamics throughout life can provide valuable information about the impact of social, environmental and biological factors on human health. The basic characteristic of motor skills is the correct and efficient execution of movements and/or the voluntary maintenance of a desired position under the influence of certain external forces and factors [3,4]. They are measurable and conditioned by genetic potential, and also under the influence of various physiological and anatomical factors, morphological characteristics, cognitive abilities, motivation and energy capacities [4]. Physical exercise can significantly influence the development and maintenance of the level of motor skills, but some motor skills can be more influenced gender and age [5,6,7,8]. Peak motor skills are most pronounced between the ages of 20 and 25, with the exception of flexibility, which peaks in childhood and motor skills are more strongly associated with body composition than with height or weight [1,4]. Significant changes have been observed in the lifestyles of young adults, including a decrease in physical activity levels, which is reflected in poorer physical performance, as well as an increase in body fat and adverse changes in physical activity [9,10]. Research indicates a decrease in physical performance, an increase in body fat percentage, and a decline in muscle mass, as well as speed and strength [11,12,13,14,15]. As these negative changes can have long-term health consequences, it is pivotal to understanding how these components develop over time.

Physical activity is essential for improving body composition and physical performance, regardless of gender, age, weight, or maturity status [16]. Consequently, university students represent a particularly relevant population for examining motor skills in relation to body composition, as this life stage is marked by significant physiological, behavioral and lifestyle transitions that can influence both parameters. Starting university is a demanding period in the life of every student. Also, many behavioral factors such as alcohol consumption, smoking, fast food, and drug usage affect the mental and physical well-being of students [17]. The change in environment and obligations during studying are often associated with a reduced level of physical activity and an increase in a sedentary lifestyle [18,19], which consequently affects the status of motor skills and body composition. Maintaining flexibility and strength in this period of life are one of the important physical predictors of quality of life in adulthood and old age [20]. Body composition assessment is carried out using anthropometric measurements and bioelectrical impedance analysis. Using various formulas, bioelectrical impedance analysis calculates the percentage of fat and lean components (water content, skeletal and smooth muscle mass, and bone mass) in the human body [21,22,23]. The above components have a great influence on strength and flexibility [24]. A higher proportion of lean mass, especially muscle tissue, is directly related to greater muscle strength. Through anthropometric data and motor ability tests, where variables are related to fat and adipose tissue, and muscle and bone development, physical activity, or physical fitness tests, show a significant difference [24].

The body composition of athletes differs in significantly higher amounts of total body water, free mass, skeletal muscle and a lower percentage of fat compared to healthy physically inactive individuals [25]. Students with a higher proportion of muscle mass show better performance in strength exercises [6]. A higher percentage of fat tissue can limit strength because it creates additional load on the body without contributing to force production. In obese individuals, strength can be reduced due to less functional muscle mass and an increased risk of injury [26]. Research shows that a higher percentage of fat tissue, especially around joints such as hips and knees, can limit flexibility due to mechanical restrictions in movement. Excessive fat in these areas can physically block the full range of motion, making it difficult to perform certain exercises or movements with full efficiency [27,28]. Also, if muscles are too tight or overtrained without adequate stretching (as can happen in strength-focused athletes), this can reduce range of motion [29]. Proper hydration is essential for maintaining muscle and connective tissue elasticity, which supports overall muscle and tendon flexibility and prevents injury [30]. Thus, while excess body fat can limit flexibility by blocking joint movement, maintaining a healthy balance of muscle mass and proper hydration can support and improve flexibility.

The aim of the study is to examine the relationship between body mass index (BMI), muscle mass and fat mass with muscle strength and flexibility in university students, as well as compare students enrolled in health study programs and those in non-health study programs. The rationale for comparing students from health-related and non-health-related study programs originates from the central research question: “Are there differences in motor and physical parameters between these two groups?” We anticipate that students pursuing careers in health-related fields may demonstrate greater intrinsic motivation to engage in health-promoting behaviors, which could translate into superior performance in physical and motor fitness assessments. Accordingly, we hypothesized that students enrolled in health study programs would exhibit more favorable motor and physical outcomes compared to students in non-health programs.

## 2. Materials and Methods

### 2.1. Body Composition Analysis

Body composition analysis was carried out using the medically certified segmental body composition analyzer TANITA MC-780MA (TANITA Corporation, Tokyo, Japan). Measurements were conducted in either the “Standard” or “Athlete” mode depending on participants’ self-reported activity levels. All individuals who are not athletes were measured in the “Standard” mode, while athletes were measured in the “Athlete” mode. The responses from athletes were verified by requiring the submission of an official certificate from the sports club in which they train and compete. Each certificate was signed and stamped by an authorized representative of the club. In accordance with the regulations of the Ministry of Sports and Tourism of the Republic of Croatia, the issuance of such a certificate confirms that the individual participates in organized sports activities at least five times per week. Taking the above into account, we have accepted the certificate as valid proof that a person is actively engaged in sports.

The device measured a range of parameters including body weight, fat mass (expressed as a percentage), muscle mass (kg), bone mass (kg), metabolic age, degree of obesity (%), total body water (TBW in kg and %), basal metabolic rate (BMR in kcal and kJ), and body mass index (BMI). All measurements were performed barefoot, with participants standing upright in a standardized position, and between 10:00 and 13:00 in the day. Participants were required to abstain from heavy meals, alcohol, stimulants (such as coffee or energy drinks), as well as excessive fluid intake prior to measurement. Instructions were provided both orally and in writing 24 h beforehand. Participants wore light, non-restrictive clothing and removed all metallic accessories.

### 2.2. Participants and Setting

The study was conducted at the University North, University Center Varaždin (UNIN2), in the Physiotherapy Laboratory 2 (FT2), between 25 October 2023, and 25 May 2024. A convenience sampling approach was employed, whereby participants were recruited from the available student population at the University North who met the inclusion criteria and voluntarily agreed to participate in the study. The final sample included 49 students from non-health-related study programs (Electrical Engineering, Civil Engineering, Multimedia and Mechanical Engineering) and 73 students from the Physiotherapy study program. The study sample consisted of both full-time and part-time university students, resulting in a naturally heterogeneous age distribution. Participants ranged in age from 18 to 44 years, with the majority (approximately 85%) falling within the 19 to 27 age range. Those who were older than 44 years, had known illnesses or injuries, or were unable to complete the full set of motor tests were excluded. In total, two participants were removed due to age criteria, and eight physiotherapy students were excluded due to incomplete motor test data related to health conditions. All eligible participants were included in the analysis to preserve transparency and reflect the diversity of the student population. Age stratification was not applied during sampling, and comparative analyses based on age groups were not conducted.

### 2.3. Motor Ability Testing

Motor abilities were tested after body composition analysis, with participants in a rested physical state. All tests were explained and demonstrated beforehand by trained and licensed physiotherapists from the Department of Physiotherapy of the University North, ensuring consistency and inter-rater reliability.

Measurements were obtained using standard instruments including a centimeter tape and a stopwatch. The motor tests were selected based on their established metric properties. All measurement instruments used in the study met recognized standards for homogeneity, sensitivity, reliability, validity and objectivity. Test administration followed standardized protocols to ensure consistency and accuracy across all assessments. The abbreviations used for motor tests are consistent with those found in established academic resources including textbooks, scientific conference materials and peer-reviewed articles in kinesiology and physiotherapy, and align with international practices for physical fitness assessment (detailed information in the Supplementary Materials).

### 2.4. Ethics and Data Protection

The study was approved by the Ethics Committee of the University of North (Class: 641-01/24-01/07, number: 2137-0336-07-24-1; date of approval: 4 April 2024). Participation was voluntary and anonymous, and all participants signed written informed consent prior to inclusion. Data privacy was maintained through encryption, and all data were stored securely on the servers of University North. Only authorized researchers had access to participant data. The study was conducted in full compliance with applicable ethical standards, ensuring confidentiality and participant welfare. There were no potential risks identified for participants, and all procedures were non-invasive and well within the scope of standard physiotherapy practice (detailed information in the Supplementary Materials).

### 2.5. Statistical Analysis

Data analysis included both descriptive and inferential statistical methods. To evaluate differences between the two student groups, the Mann–Whitney U test was applied for continuous, non-normally distributed variables (such as body composition indicators), while the chi-square test was used to assess associations between categorical variables. Relationships between variables were assessed using Spearman’s rank correlation coefficient (r), ranging from −1 to +1. Multiple linear regression analysis was performed to examine the effect of independent variables on selected outcomes. The Kruskal–Wallis test was used to compare differences between groups, chosen due to its non-parametric nature. Statistical significance was set at an alpha level of 0.05 (two-tailed), representing a 95% confidence interval. All analyses were conducted using IBM SPSS Statistics for Windows, Version 26.0 (IBM Corp., Armonk, NY, USA).

## 3. Results

A total of 122 respondents participated in the study, of which 73 (59.8%) were health students and 49 (40.2%) were non-health students. According to the gender distribution of the total 122 respondents, 56 (45.9%) were male, while 66 (54.1%) were female. Furthermore, the arithmetic mean of the respondents’ age is 20.02 with a standard deviation of 3.946, with the minimum value being 18, while the maximum value is 44.

Using the Mann–Whitney U test, statistically significant differences between health students and non-health students were found in total fat percentage (*p* < 0.001), total fat mass (kg) (*p* = 0.007), basal metabolic rate (*p* = 0.004), fat-free mass (*p* = 0.001), protein muscle mass (*p* = 0.001), protein mass percentage (*p* < 0.001), skeletal muscle mass (kg) (*p* = 0.002), skeletal muscle mass percentage (*p* = 0.001), bone mass (*p* = 0.001), sarcopenia index (*p* < 0.001), total body water percentage (*p* < 0.001), total body water (kg) (*p* = 0.003), extracellular water (*p* = 0.010), ratio of extracellular water to total body water (*p* = 0.011), intracellular water (*p* = 0.002), ratio of intracellular water to total body water (*p* = 0.004), phase angle (bioelectrical impedance) (*p* = 0.004), trunk fat percentage (*p* = 0.001), trunk fat mass (kg) (*p* = 0.014), left arm fat percentage (*p* < 0.001), left arm fat mass (kg) (*p* = 0.003), left leg fat percentage (*p* < 0.001), left leg fat mass (kg) (*p* = 0.008), right arm fat percentage (*p* < 0.001), right arm fat mass (kg) (*p* = 0.003), right leg fat percentage (*p* = 0.001), right leg fat mass (kg) (*p* = 0.013), trunk protein muscle mass (*p* = 0.001), left arm protein muscle mass (*p* < 0.001), left leg protein muscle mass (*p* = 0.002), right arm protein muscle mass (*p* < 0.001), and right leg protein muscle mass (*p* = 0.002). In the majority of these indicators, students in non-health-related programs showed higher values, suggesting greater fat and muscle mass distribution compared to their health-studies counterparts.

**Figure 1:**
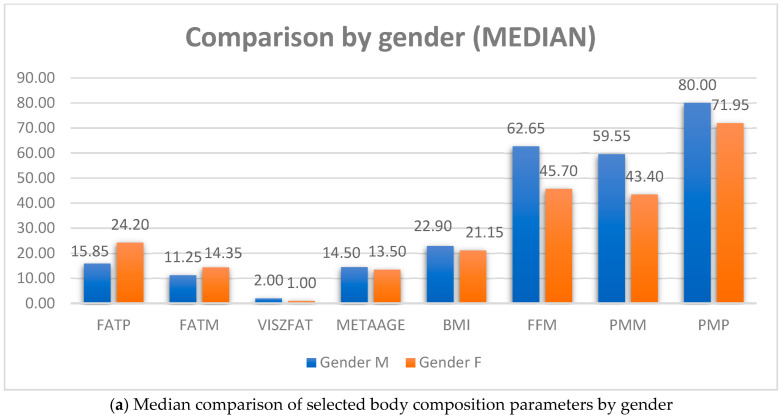

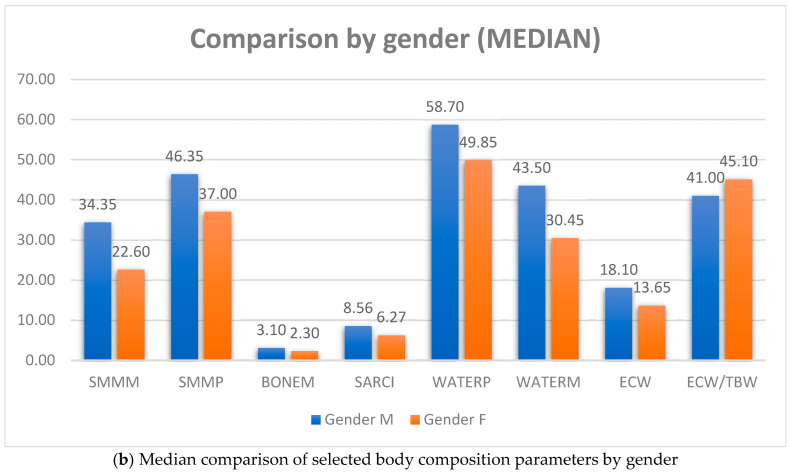
Median comparison of selected body composition parameters by gender.

However, no statistically significant differences were observed in body mass index (*p* = 0.987), visceral fat (*p* = 0.961), total body weight (*p* = 0.269) or metabolic age (*p* = 0.260), indicating that overall body size and metabolic age were comparable across both groups. Additionally, a chi-square test evaluating the association between study program and body mass index categories showed no statistically significant difference (χ^2^ = 3.003, df = 3, *p* = 0.391), suggesting that the distribution of individuals across underweight, normal weight, overweight and obese categories did not differ significantly between students of health and non-health programs. According to the gender of the respondents, a statistically significant difference (*p* < 0.05) was observed for all variables except for the variables METAAGE. A comparison of medians is shown in Figure 1a,b.

Both, positive and negative correlations were recorded between the observed variables. The highest significant negative correlations were recorded between the indicators: body fat in percentage (TRFATP) and repetitive strength—lifting the body from a lying position (MRSPTL) (r = −0.547; *p* < 0.01), metabolic age (METAAGE) and MRSPTL (r = −0.444; *p* < 0.01) while the highest significant positive correlation was recorded between the indicators:, and body fat in kilograms (TRFATM) (r = 0.941; *p* < 0.01 (TRFATM) and METAAGE (r = 0.939; *p* < 0.01) presented in Table 1.

**Figure 2:**
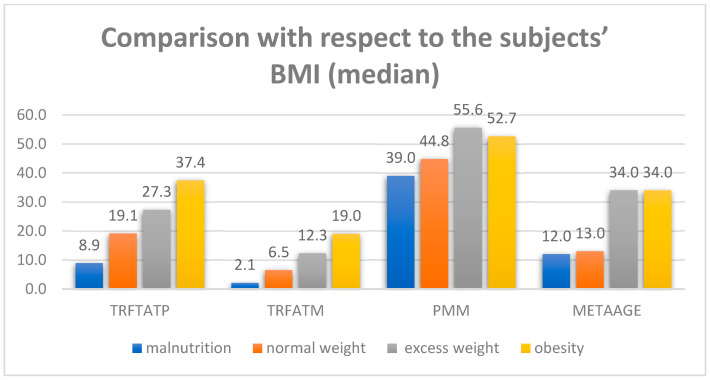
Median comparison of body composition parameters across BMI categories.

If we look at the significance value for all observed variables, it can be seen that *p* is less than 0.05, so it can be said, with a confidence level of 95%, that there is a statistically significant difference with respect to the BMI of the subjects. According to the distribution, in the health studies group we had 5 (71.4%) Malnutrition, 49 (59%) Normal, 13 (52%) Overweight and 6 (85.7%) Obese. In non-health students this distribution was: 2 (28.6%) Malnutrition, 34 (41%) Normal, 12 (48%) Overweight and 1 (14.3%) Obese. Comparison of BMI across studies did not show statistical significance. In Figure 2, it can be seen that the median value is higher for subjects with a higher BMI value (overweight and obesity).

Table 2 shows the results of multiple linear correlation with the dependent variable (criterion) MRSPTL and predictors: TRFATP, TRFATM, muscle mass in kilograms (PMM), METAAGE. The prediction model explains 44.5% of the variance of the criteria. In this case, higher values for MRSPTL are found in subjects with higher response values for PMM (β = 0.565, *p* < 0.05) and lower values for TRFATM (β = −0.858, *p* < 0.05), and the model is statistically significant (*p* < 0.05). From the aforementioned Table 3, it can be seen that positive and negative correlations were recorded between the observed variables, the highest significant negative correlations were recorded between the indicators: TRFATP and MFLPRR (r = −0.467; *p* < 0.01) and METAAGE and MFLPRR (r = −0.476; *p* < 0.01), while the highest significant positive correlation was recorded between the indicators: TRFATM and TRFATP (r = 0.976; *p* < 0.01), TRFATP and METAAGE (r = 0.886; *p* < 0.01).

From Table 3 presented above, it can be seen that there was a positive and negative correlation between the observed variables, the highest significant negative correlations were recorded between the indicators: TRFATP and MFLPRR (r = −0.467; *p* < 0.01) and METAAGE and MFLPRR (r = −0.476; *p* < 0.01), while the highest significant positive correlation was recorded between the indicators: TRFATP and TRFATM (r = 0.976; *p* < 0.01), TRFATP and METAAGE (r = 0.886; *p* < 0.01). The prediction model explains 34.9% of the variance of the criteria. In this case, a higher value for MRSPTL was found in respondents with a higher response value for PMM (β = 0.643, *p* < 0.01) and a lower value for METAAGE (β = −0.911, *p* < 0.05), the model is statistically significant (*p* < 0.05).

The prediction model in Table 4 explains 34.9% of the variance of the criteria. In this case, higher values for MRSPTL are found in respondents with higher response values for PMM (β = 0.643, *p* < 0.01) and lower values for METAAGE (β = −0.911, *p* < 0.05), and the model is statistically significant (*p* < 0.05).

## 4. Discussion

Our study found notable differences in body composition between students from health and non-health-related programs, with the latter group exhibiting generally higher values in fat mass, muscle mass, total body water and segmental body measurements. Although the distribution of BMI categories was similar across both groups, the detailed analysis of body composition reveals distinct physiological profiles that may reflect differences in lifestyle, physical activity levels or even academic demands. These disparities suggest that body composition may be influenced by factors beyond BMI alone, emphasizing the importance of examining fat and muscle distribution when assessing overall health and fitness among student populations. Also, BMI is not sufficient as an indicator of health, given the clear differences in body composition despite similar BMI values. Negative correlations of estimated values of motor abilities of repetitive strength and flexibility between the values of body fat percentage and metabolic age were also recorded in both groups (Table 1 and Table 3). An optimal body mass index enables easier performance of motor tasks [24,31,32,33], and well-developed motor abilities aid in improving health and achieving normal body weight [16,34]. Their interrelation can directly affect the prevention of chronic diseases, functional abilities, social inclusion, development of interpersonal skills, as well as other fundamental aspects related to quality of life [35,36,37,38].

The largest proportion of participants from both groups is in the normal body mass category. Similar results in the distribution of body mass index in the student population were also recorded in the study by Zaccagni et al. [39]. The results of the study by de Faria Filha et al. [40], conducted in 2018 on 2245 health students in Brazil, indicate the prevalence of overweight (48.3%) and obesity (15.3%) in their study population, while in our study this figure for overweight students is 20%, and for obese students it is 6%.

The results also indicate clear differences in the body composition of the participants depending on the body mass index. In both groups, increased values of the observed parameters. The percentage of body fat and absolute fat mass progressively increase with increasing body mass index, reaching the highest values in the obesity category, which is in line with the findings of Schutter et al. [41]. These findings indicate an unfavorable effect of an increased body mass index on body composition, with an emphasis on the accumulation of fat tissue [42]. For optimal motor abilities, it is important to maintain a healthy proportion of fat and muscle mass, since this allows for better performance in most physical activities [43]. Muscle mass shows a different pattern, with the highest proportion of muscle mass being recorded in overweight individuals. Reduced muscle mass can also be found in students with malnutrition and/or obesity, suggesting that an increase in body mass index above a certain threshold is not associated with further increases in muscle mass [44]. Also, metabolic age is significantly higher in students in both overweight and obese groups compared to normal weight and malnutrition. The results obtained did not show a statistically significant difference between the groups in bioimpedance scale parameters and performance of motor skills requiring flexibility. Motor deficits in overweight individuals are most pronounced in motor abilities that require lifting one’s own body against gravity [31,45]. The results of the study by Altavilla et al., [46] and Haddad et al. [47] also indicate a clear link between BMI and motor performance. Participants with a normal index showed significantly better motor abilities compared to participants with higher body mass values. The aforementioned studies also confirm that an increased index can negatively affect motor performance due to reduced strength and endurance [46,47], while other studies describe the negative effect associated with increased body fat on motor abilities as a developmental disorder of lack of coordination during movement [31,48,49,50].

The results of the study indicate that there are statistically significant differences in the motor abilities of students depending on their level of nutrition and BMI. They also highlight the negative impact of an elevated body mass index on body composition and metabolic parameters—and suggest the importance of maintaining optimal body weight in order to reduce the risk of metabolic and functional disorders. An increase in the proportion of body fat can negatively affect muscle strength and endurance, balance and coordination, and can also increase the load on the joints, which can lead to poorer motor performance [51,52]. Also, in addition to reduced and poor motor abilities, it is important to note that negative effects can also extend to influence the perception of personal psychophysical state. A reduced level of the aforementioned abilities can also negatively affect the decision of young individuals to participate in physical and/or sports activities. Understanding the aforementioned relationship between overweight/obesity and motor abilities, along with psychophysical characteristics, can aid researchers in developing effective strategies by integrating various psychophysical variables related to the needs of the participants. While performing physical activity, an individual stimulates the development of their intellectual abilities, builds their personality, and acquires social skills necessary for coexistence in the community [41,53]. Several limitations of our study should be acknowledged. The relatively small sample size due to convenience sampling approach, especially when divided into subgroups based on educational program and BMI categories, limits the generalizability of our findings. There is also the issue of the inclusion of a small number of older participants, which, while intended to ensure transparency and reflect the broader university population in Croatia, could potentially introduce some variability in the results; however, given their low representation, this is unlikely to have significantly affected the overall findings. Furthermore, the cross-sectional design of the study does not allow for establishing causality between body composition and motor abilities, and self-reported physical activity levels were not analyzed. Then, body composition was assessed using bioelectrical impedance analysis, which (albeit practical and non-invasive) may be influenced by hydration status and other external factors, potentially affecting measurement accuracy. Additionally, factors such as physical activity levels, dietary habits or socio-economic status can have an impact on both body composition and motor performance, but they were not controlled for in this study. Another limitation is the absence of physiological measures such as resting heart rate and blood pressure, which are important indicators of physical fitness and could have provided additional context to the body composition and motor performance data. Finally, the voluntary nature of participation may introduce a type of selection bias, as students more interested or engaged in health-related behaviors may have been more likely to participate.

## 5. Conclusions

This pilot study demonstrated statistically significant differences in body composition and motor abilities among students, influenced by their BMI and nutritional status. Students with higher fat tissue levels and elevated BMI showed reduced motor performance, particularly in strength and flexibility tests. These physical limitations may also negatively impact the perception of personal psychophysical well-being, potentially discouraging engagement in physical activities.

Given that the student population represents a critical developmental period for establishing long-term health behaviors, the results emphasize the importance of early identification and intervention. Preventive strategies could be focused on promoting regular physical activity, balanced nutrition, and awareness of their impact on health and quality of life. Further research on larger, more diverse samples (and by using longitudinal studies) will be needed to better understand these associations and support the development of effective interventions for students.

## Figures and Tables

**Table 1:** Spearman correlation coefficient I (group of health students—73).

	1	2	3	4	5	6	7	8	9
1. WEIGHT	1.000								
2. TRFATP	0.532 **	1.000							
3. TRFATM	0.755 **	**0.** 941 **	1.000						
4. PMM	0.829 **	0.025	0.321 **	1.000					
5. METAAGE	0.681 **	0.897 **	0.939 **	0.274 *	1.000				
6. MFLPRR	−0.189	−0.198	−0.225	−0.090	−0.295 *	1.000			
7. MRSPTL	−0.004	−0.547 **	−0.402 **	0.351 **	−0.444 **	0.195	1.000		
8. MRSNK	0.107	−0.503 **	−0.325 **	0.486 **	−0.285 *	−0.046	0.570 **	1.000	
9. MRPLČ	0.366 **	−0.109	0.047	0.544 **	−0.010	0.020	0.348 **	0.353 **	1.000

** Correlation is significant at the 0.01 level (2-tailed). * Correlation is significant at the 0.05 level (2-tailed).

**Table 2:** Regression analysis with respect to the dependent variable MRSPTL (group of health students—73).

	β	*t*	*p*	Model Summary
TRFTATP	0.380	0.932	0.355	corrected R^2^ = 0.445 F(4.68) = 15.419 *p* < 0.001
TRFATM	−0.858	−2.007	**0.049**	
PMM	0.565	4.089	**0.000**	
METAAGE	−0.173	−0.851	0.398	

Legend: β = value of standardized regression coefficient; t = *t*-test value; *p* = significance level; adjusted R^2^ = adjusted total contribution to explained variance; F = value of total F-ratio.

**Table 3:** Spearman correlation coefficient II (group of non-health students—49).

	1	2	3	4	5	6	7	8	9
1. WEIGHT	1.000								
2. TRFATP	0.600 **	1.000							
3. TRFATM	0.710 **	0.976 **	1.000						
4. PMM	0.877 **	0.211	0.363 *	1.000					
5. METAAGE	0.641 **	0.886 **	0.874 **	0.323 *	1.000				
6. MFLPRR	−0.156	−0.467 **	-0.425 **	−0.006	−0.476 **	1.000			
7. MRSPTL	0.186	−	−	0.409 **	−0.298 *	0.129	1.000		
8. MRSNK	0.174	−0.259	−0.211	0.407 **	−0.232	0.102	0.779 **	1.000	
9. MRPLČ	0.051	−0.088	−0.078	0.132	−0.173	0.030	0.612 **	0.511 **	1.000

** Correlation is significant at the 0.01 level (2-tailed). * Correlation is significant at the 0.05 level (2-tailed).

**Table 4:** Regression analysis with respect to the dependent variable MRSPTL (group of non-health students—49).

	β	*t*	*p*	Model Summary
TRFTATP	0.482	1.374	0.176	corrected R^2^ = 0.349 F(4.44) = 7.444 *p* < 0.001
TRFATM	−0.073	−0.156	0.877	
PMM	0.643	3.728	**0.001**	
METAAGE	−0.911	−3.151	**0.003**	

Legend: β = value of standardized regression coefficient; t = *t*-test value; *p* = significance level; adjusted R^2^ = adjusted total contribution to explained variance; F = value of total F-ratio.

## Data Availability

The data provided in this study can be obtained upon request from the corresponding author.

## Supplementary Materials

The following supporting information can be downloaded at: https://www.mdpi.com/article/10.3390/life15101504/s1. Table S1. Tests of Normality **(Z)**. Table S2. Tests of Normality **(NZ)**. Table S3. Comparison with respect to the sex of respondents. Table S4. Comparison with respect to the sex of respondents. Table S5. Coefficients ^a^ (dependent). Table S6. Coefficients ^a^ (independent). Table S7. z tests—correlations: Two independent Pearson r’s. Figure S1. Power analysis.
