# Supplementary material for: Motor Capabilities and Body Composition in Health vs. Non-Health University Students: A Pilot Study
Source: Life (Basel). 2025 Sep 24;15(10):1504. doi: 10.3390/life15101504 (PMC12565337; doi:10.3390/life15101504)
Supplement: Supplementary file 1 [file life-15-01504-s001.zip › life-3735221-supplementary.pdf]

Supplementary Materials

Motor Capabilities and Body Composition in Health vs. Non-Health University Students: A Pilot Study – Appendix

Ivana Živoder[1*], Vesna Hodić[1], Nikolina Zaplatić Degač[1], , Jasminka Potočnjak[1], Marija Arapović[1], Anica Kuzmić[1], Željko Jelec[2], Goran Knežević[3], Mateja Znika[1], Tomislav Meštrović[1]

1 University North, 42000 Varazdin, Croatia: ivana.zivoder@unin.hr; vesna.hodic@unin.hr; nzaplatic@unin.hr; marapovic@un.hr; akuzmic@unin.hr; jpotocnjak@unin.hr; mznika@unin.hr; tmestrovic@unin.hr;
2 Special hospital St. Katarina, 10000 Zagreb, Croatia; zjelec@yahoo.com
3 StatVall, 33000 Virovitica, Croatia; statistika.analiza@gmail.com
* Correspondence: ivana.zivoder@unin.hr

1. Description of abbreviations

Abbreviation	Explanation
FATP	body fat in percentage
FATM	body fat in kilograms
PMM	protein muscle mass
METAAGE	metabolic age
PMP	protein mass percentage
SMM	skeletal muscle mass
SMMP	skeletal muscle mass percentage
FFM	fat-free mass
WATER P	total body water percentage
WATER M	total body water (kg)
ECW	extracellular water
ECW/TBW	ratio of extracellular water to total body water
SARCI	sarcopenia index
BONE M	bone mass
BMI	body mass index
MFLISK	flexibility of the shoulder girdle
MFLPRG-D, MFLPRG-N	flexibility of the dominant and non-dominant arm
MFLPRR	trunk flexibility in a wide-leg position
MBFTAP	speed evaluated through a hand tapping test
MAGKUS	coordination via lateral step agility
MRSPTL	repetitive strength – lifting the body from a lying position
MRSNK	push-ups on the knees
MRPLČ	half-squats

2. Description of motor ability tests

MFLISK – turn / flexibility of the shoulder girdle

FL – Flexibility
The prop is a round stick with a diameter of 2.5 cm, 165 cm long, at one end of the stick, there is a plastic holder 15 cm long, on which a centimeter strip with a zero point is drawn along the stick along the edge of the plastic holder
Task:
- the subject raises the stick in an upturned position with outstretched arms, making a turn, in such a way that the "weaker" hand holds the stick, and the "better" hand slides the stick, separating the hands so that the turn is made with the smallest possible distance between the hands
Note:
- the task is demonstrated, but there are no trial attempts
- the turn must be performed slowly and without swinging
- the shoulders must be turned simultaneously, with outstretched arms
- the task is performed at least three times without a break
- the distance between the inner edges of the hands is measured

MFLPRR – trunk flexibility in a wide-leg position / forward bend with different legs
Description:
- two lines are drawn in front of the wall 2 m at an angle of 45°, so that the tip of the angle touches the wall, and the sliding meter is placed perpendicular to the wall
Starting position:
- the subject sits cross-legged on the ground with his back and head against the wall
- he/she spreads his extended legs so that his feet lie on the marked lines
- he/she extends his arms, overlapping his middle fingers, and lowers them to the ground in front of him (the zero point of the meter is set here)
Task:
- is to do a deep forward bend, with his legs and arms extended, sliding along the meter on the ground, without jerking
Note:
- the task is demonstrated, but there are no trial attempts
- it is performed at least 3 times without a break
- there are no sudden jerks during the test
- it is recorded in centimeters

MBFTAP – hand tapping
BF – Speed of Movement Frequency
Description:
- we place a taping board on the table, and in front of it we place a chair. The board is 1 m long, 25 cm wide, and 1-2 cm high. Two round plates with a diameter of 20 cm are attached to the board, at a distance (within the inner edges) of 61 cm
Task:
- the subject places the palm of the "weaker" hand in the middle of the board, and places the "better" hand crosswise just above the round part of the board
- at the sound signal, the subject should touch one round plate and then the other as quickly as possible with the "better" hand within 15 seconds
Note:
- the minimum dimensions of the measuring space are 2 x 2m
- the task is demonstrated, but the subject does not have trial attempts
- the task is measured at least 3 times, with a sufficiently long pause
- incorrect touches are when the subject: hits the round part of the board more than 1 time, misses the round part of the board, hits "too softly", or does not perform the hits alternately.

MRPLČ – half-squat test
Equipment: Stopwatch, school chair.
Description: The subject stands in front of a chair with his back to the chair and at a distance slightly shorter than his upper thigh. Feet is placed parallel, shoulder-width apart, and hands are in front. On the signal "now", the subject performs as many lowerings (half-squats) as possible from the starting position to the level of the chair and back to the starting position, within 30 seconds. The examiner is located one meter away from the subject, controls the performance, and counts the correctly performed half-squats.
Scoring: The result is the number of correctly performed half-squats within 30 seconds. The test is performed once.
Note: The test is modified according to the age of the subject. For the entire duration of the test, the hands must be in front. The subject should reach the level of the chair only with the tip of the buttocks.
The task is demonstrated while giving instructions to the subject. The subject has the right to a trial attempt to determine the adequate distance of the feet from the chair required for the correct performance of the test.

MRSPTL – trunk curl-up (sit-Up) test of repetitive strength – lifting the body from a lying position
Purpose: Assessment of the repetitive strength of the anterior trunk (abdominal muscles).
Equipment: Exercise mat, stopwatch.
Procedure: The participant lies on their back on a mat, knees bent at approximately 90°, feet flat on the floor, and arms crossed over the chest (or palms on thighs if required by protocol).
At the "Go" signal, the participant performs as many proper trunk lifts (curl-ups/sit-ups) as possible within 30 seconds.
Each repetition requires the shoulder blades to lift off the floor until elbows (or hands, depending on the version) pass the knees/thighs, then return to the starting position with the back touching the mat.The score is the number of correctly executed repetitions in 30 seconds.
Key points: Feet remain flat on the floor, no jerking or using arms for momentum, maintain controlled pace.

MRSNK – push-ups on the knees
Purpose: Assessment of repetitive strength of the arms and shoulder girdle.
Equipment: Exercise mat, stopwatch.
Procedure: The participant starts in a plank position with knees on the mat, hands slightly wider than shoulder-width, body aligned from shoulders to knees.
At the "Go" signal, the participant lowers the chest toward the floor until the elbows reach approximately 90° (upper arms parallel to the floor), then pushes back to full arm extension. Perform as many correct push-ups as possible within 30 seconds. The score is the number of proper repetitions in 30 seconds.
Key points: Keep the body straight (no sagging or arching), lower and raise in a controlled manner, full range of motion required for each repetition

MAGKUS – side-step (lateral agility) test
Purpose: To assess lateral agility and speed, measuring how quickly the participant can move sideways over a set distance.
Equipment: space: a flat, firm surface at least 6 × 3 m (indoors).
Markings: on the floor, mark two parallel lines, each 1 m long and 4 m apart.
Timing device: stopwatch accurate to 0.01 s.

Procedure: starting position; the participant stands upright with feet together (parallel) inside the lines, positioned laterally next to the first line.
The body remains sideways throughout the test.
Movement: at the command "Go", the participant moves as quickly as possible laterally (side-step) toward the opposite line, without crossing the feet (no grapevine or crossover steps). Upon stepping across the second line with the outside foot, the participant stops briefly and, without turning the body, immediately moves back toward the first line using the same side-step technique.
Repetitions: the participant repeats this back-and-forth movement until they have crossed the 4-meter gap a total of six times (i.e., three full round trips). The task is complete when the participant touches or crosses the final line with the outside foot on the sixth crossing.
Timing: the stopwatch starts on the "Go" signal and stops the moment the participant touches or crosses the final line after the sixth crossing. Time is recorded in hundredths of a second (0.01 s).
Scoring: the result is the fastest time (in seconds) achieved over three trials. There is a 1-minute rest between each attempt.
Key Points / Rules: maintain a sideways body position at all times. Feet must not cross during side-stepping. Each line must be touched or crossed with the outside foot for the repetition to count. Movement should be quick but controlled to ensure safety and accuracy.

3. Testing protocol

1. Anthropometry and body composition – measurement on an impedance scale (while adhering to instructions related to hydration, meals, energy drinks and other stimulants).

2. Motor tests – the measuring instruments used in the study meet the standards of homogeneity, sensitivity, reliability, validity and objectivity. We adhered to the specific instructions for each motor test. Prskalo and Sporiš (2016) define metric characteristics as prerequisites, i.e., standards and protocols that make a measuring instrument suitable for certain measurements (source: Prskalo I, Halačev S, editors. Kinesiology. Zagreb: Školska knjiga; Faculty of Teacher Education, University of Zagreb; Faculty of Kinesiology, University of Zagreb; 2016).

Sequence of motor tests

**A)** MFLISK	flexibility of the shoulder girdle
**B)** MFLPRG	flexibility of the dominant and non-dominant arm
**C)** MFLPRR	trunk flexibility in a wide-leg position
**D** MBFTAP	speed evaluated through a hand tapping test
**E)** MAGKUS	coordination via lateral step agility
**F)** MRSPTL	repetitive strength – lifting the body from a lying position
**G)** MRSNK	push-ups on the knees
**H)** MRPLČ	half-squats

4. Additional statistical analyses

In the following section, the distribution of the observed variables was examined through tests of normality, specifically the Kolmogorov-Smirnov and Shapiro-Wilk procedures. The purpose of these tests was to establish whether the data followed a normal (Gaussian) distribution, which represented a key assumption for the application of many statistical techniques. Determining the distributional characteristics of the variables was essential, as it directly influenced the choice of subsequent analytical methods. If the assumption of normality was met ($p \geq 0.05$), parametric tests were applied,

given their greater statistical power under these conditions. However, if normality was violated ($p < 0.05$), nonparametric alternatives were employed, as they did not rely on this assumption and were more robust in handling skewed or irregularly distributed data.

In this study, tests of normality were systematically conducted for all variables under investigation. This ensured that the analytical strategy rested on adequate methodological grounds and that the choice between parametric and nonparametric approaches was made transparently and in line with the empirical properties of the dataset. Presenting these results allowed for a clearer interpretation of the findings and provided readers with an explicit rationale for the statistical methods selected in subsequent analyses.

Table S1: Tests of Normality **(Z)**

	Kolmogorov-Smirnov[a]			Shapiro-Wilk		
	Statistic	df	Sig.	Statistic	df	Sig.
WEIGHT	,083	73	,200*	,965	73	,040
TRFATP	,091	73	,200*	,981	73	,324
TRFATM	,146	73	,001	,929	73	,000
PMM	,103	73	,051	,933	73	,001
METAAGE	,245	73	,000	,794	73	,000
MFLPRR	,059	73	,200*	,991	73	,901
MRSPTL	,104	73	,050	,965	73	,040
MRSNK	,147	73	,001	,965	73	,042
MRPLČ	,120	73	,011	,906	73	,000

*. This is a lower bound of the true significance.

a. Lilliefors Significance Correction

From the significance values presented in Table S1, it was possible to observe the outcomes of the Kolmogorov-Smirnov and Shapiro-Wilk tests of normality. Specifically, when the significance value for a given variable exceeded 0.05 ($p > 0.05$), the data were considered to follow a normal distribution, whereas significance levels below 0.05 indicated that the distribution deviated from normality. Since, for a considerable portion of the variables, the significance levels did not surpass the 0.05 threshold, it was concluded that the assumption of normality was not met in all cases. Consequently, further analyses were carried out using nonparametric tests, as these methods are more suitable and statistically valid when dealing with data that do not conform to the requirements of parametric testing.

Table S2: Tests of Normality **(NZ)**

	Kolmogorov-Smirnov[a]			Shapiro-Wilk		
	Statistic	df	Sig.	Statistic	df	Sig.
WEIGHT	,106	49	,200*	,959	49	,088
TRFATP	,112	49	,164	,924	49	,004
TRFATM	,157	49	,004	,905	49	,001
PMM	,072	49	,200*	,975	49	,370
METAAGE	,307	49	,000	,737	49	,000
MFLPRR	,084	49	,200*	,969	49	,224
MRSPTL	,124	49	,057	,983	49	,689
MRSNK	,170	49	,001	,940	49	,015
MRPLČ	,096	49	,200*	,975	49	,388

*. This is a lower bound of the true significance.

a. Lilliefors Significance Correction

From the significance values presented in Table S2, the results of the Kolmogorov-Smirnov and Shapiro-Wilk tests of normality can be interpreted. As with the previous table, if the significance value for a given variable was greater than 0.05 ($p > 0.05$), the data were considered normally distributed, whereas values below 0.05 indicated a departure from normality. Since for a substantial share of the variables the significance level did not exceed the 0.05 threshold, it was concluded that the assumption of normality was not satisfied in all cases. Therefore, the subsequent statistical analyses were conducted using nonparametric methods, which provide a more appropriate and reliable approach when the requirements for parametric testing are not fully met.

Table S3: Comparison with respect to the sex of respondents

		SEX	
		M	F
FATP	Median	15,85	24,20
	Percentile 25	11,80	19,10
	Percentile 75	23,00	32,50
FATM	Median	11,25	14,35
	Percentile 25	8,95	10,60
	Percentile 75	18,60	21,80
VISZFAT	Median	2,00	1,00
	Percentile 25	1,00	1,00
	Percentile 75	5,00	2,00
METAAGE	Median	14,50	13,50
	Percentile 25	12,00	12,00
	Percentile 75	30,50	30,00
BMI	Median	22,90	21,15
	Percentile 25	21,30	19,40
	Percentile 75	25,50	24,50
FFM	Median	62,65	45,70
	Percentile 25	60,10	42,10
	Percentile 75	69,50	50,40
PMM	Median	59,55	43,40
	Percentile 25	57,10	40,00
	Percentile 75	66,05	47,80
PMP	Median	80,00	71,95
	Percentile 25	73,20	64,10
	Percentile 75	83,85	76,80

Table S4: Comparison with respect to the sex of respondents

		SEX	
		M	F
SMMM	Median	34,35	22,60
	Percentile 25	33,30	21,60
	Percentile 75	37,45	23,70
SMMP	Median	46,35	37,00
	Percentile 25	42,70	34,30
	Percentile 75	48,90	39,20
BONEM	Median	3,10	2,30
	Percentile 25	3,00	2,10

	Percentile 75	3,45	2,60
SARCI	Median	8,56	6,27
	Percentile 25	7,99	5,97
	Percentile 75	9,07	6,49
WATERP	Median	58,70	49,85
	Percentile 25	54,05	46,90
	Percentile 75	61,35	52,70
WATERM	Median	43,50	30,45
	Percentile 25	42,50	28,70
	Percentile 75	47,40	32,20
ECW	Median	18,10	13,65
	Percentile 25	17,20	12,60
	Percentile 75	19,25	14,90
ECW/TBW	Median	41,00	45,10
	Percentile 25	40,30	43,30
	Percentile 75	41,70	46,40

Table S5: Coefficients[a] (dependent)

Model		Unstandardized Coefficients		Standardized Coefficients			95,0% Confidence Interval for B	
		B	Std. Error	Beta	t	Sig.	Lower Bound	Upper Bound
1	(Constant)	18,722	10,228		1,830	,072	-1,687	39,131
	TRFATP	,490	,525	,380	,932	,355	-,558	1,537
	TRFATM	-1,957	,975	-,858	-2,007	,049	-3,903	-,011
	PMM	,636	,156	,565	4,089	,000	,326	,947
	METAAGE	-,191	,225	-,173	-,851	,398	-,640	,257

a. Dependent Variable: MRSPTL - LIFTING THE BODY FROM A LYING POSITION – THE NUMBER OF REPETITIONS IN 60 SECONDS

Table S6: Coefficients[a] (independent)

Model		Unstandardized Coefficients		Standardized Coefficients			95,0% Confidence Interval for B	
		B	Std. Error	Beta	t	Sig.	Lower Bound	Upper Bound
1	(Constant)	23,777	8,032		2,960	,005	7,590	39,965
	TRFATP	,458	,333	,482	1,374	,176	-,214	1,130
	TRFATM	-,142	,913	-,073	-,156	,877	-1,983	1,698
	PMM	,518	,139	,643	3,728	,001	,238	,799
	METAAGE	-,821	,261	-,911	-3,151	,003	-1,346	-,296

a. Dependent Variable: MRSPTL - LIFTING THE BODY FROM A LYING POSITION – THE NUMBER OF REPETITIONS IN 60 SECONDS

Table S7: z tests - correlations: Two independent Pearson r's

**Analysis:**	A priori: Compute required sample size	
**Input:**	Tail(s) = Two	
	Effect size q = 0.6	
	α err prob = 0.05	
	Power (1-β err prob)	= 0.80
	Allocation ratio N2/N1 = 1	
**Output:**	Critical z = 1.9599640	
	Sample size group 1 = 47	
	Sample size group 2 = 47	
	Total sample size = 94	
	Actual power = 0.8035275	

An a priori power analysis was conducted using the Compute required sample size' procedure. The subsequent testing was performed with z tests for correlations, specifically comparing two independent Pearson r's. Based on an assumed effect size of q = 0.6, an alpha error probability of 0.05, and a statistical power of 0.80, the analysis indicated that a minimum total sample of 94 participants was required, corresponding to two groups of 47 participants each.

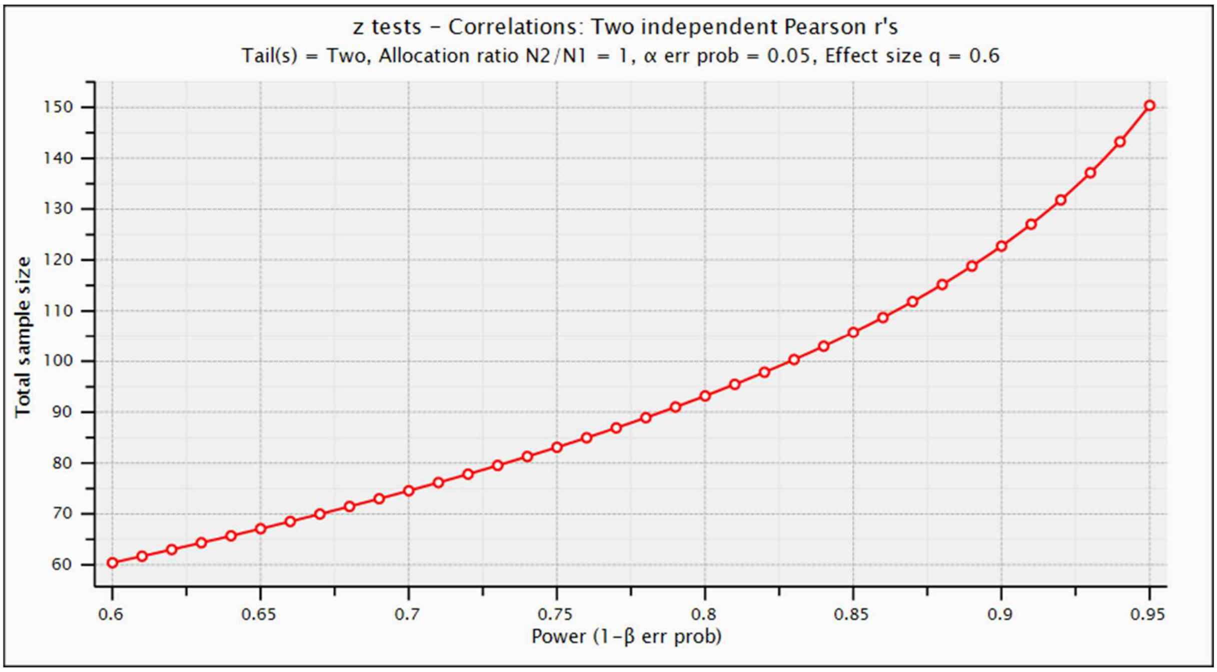

Figure S1. Power analysis

Author Contributions: Conceptualization, I. Z. and V. H.; methodology, V. H.; software, G. K.; validation, G. K., N. Z. D. and M. A.; formal analysis, G. K.; investigation, N. Z. D., V. H., M. A., T. M.; resources, M. Z.; data curation, I. Z..; writing—original draft preparation, I. Z., N. Z. D., J. P., A, K., M. A. and T.M.; writing—review and editing, M. Z., T.M.; visualization, V. H.; supervision, I. Z., Z. J.; project administration, M. A.; funding acquisition, Z. J. All authors have read and agreed to the published version of the manuscript.

Funding: This research received no external funding. The authors received financial support for the research by University North: Grant for Scientific Research and Artwork of the University North – Biomedicine (Area of Science).

Institutional Review Board Statement: The study was conducted in accordance with the Declaration of Helsinki and approved by the Institutional Ethics Committee of University North (CLASS: 641-01/24-01/07 NUMBER: 2137-0336-07-24-1; Date of approval: April 4, 2024) for studies involving humans.

Informed Consent Statement: Written informed consent was obtained from all subjects involved in the study.

Data Availability Statement: The data provided in this study can be obtained upon request from the corresponding author.

Acknowledgements: The authors wish to thank the students at University North for participating during the research period.

Conflicts of Interest: The authors declare no conflicts of interest.
